# An efficient method for gene silencing in human primary plasmacytoid dendritic cells: silencing of the TLR7/IRF-7 pathway as a proof of concept

**DOI:** 10.1038/srep29891

**Published:** 2016-07-14

**Authors:** Nikaïa Smith, Pierre-Olivier Vidalain, Sébastien Nisole, Jean-Philippe Herbeuval

**Affiliations:** 1 Equipe Chimie et Biologie, Modélisation & Immunologie pour la Thérapie (CBMIT), CNRS UMR8601, Laboratoire de Chimie et de Biochimie Pharmacologiques et Toxicologiques, CICB-Paris (FR 3567), Centre Universitaire des Saints-Pères, 45 rue des Saints Pères, 75006, Paris, France; 2 Université Paris Descartes, Sorbonne Paris Cité, Paris, France; 3 INSERM UMR-S 1124, 45 rue des Saints-Pères, 75006 Paris, France

## Abstract

Plasmacytoid dendritic cells (pDC) are specialized immune cells that produce massive levels of type I interferon in response to pathogens. Unfortunately, pDC are fragile and extremely rare, rendering their functional study a tough challenge. However, because of their central role in numerous pathologies, there is a considerable need for an efficient and reproducible protocol for gene silencing in these cells. In this report, we tested six different methods for siRNA delivery into primary human pDC including viral-based, lipid-based, electroporation, and poly-ethylenimine (PEI) technologies. We show that lipid-based reagent DOTAP was extremely efficient for siRNA delivery into pDC, and did not induce cell death or pDC activation. We successfully silenced Toll-Like Receptor 7 (TLR7), CXCR4 and IFN regulatory factor 7 (IRF-7) gene expression in pDC as assessed by RT-qPCR or cytometry. Finally, we showed that TLR7 or IRF-7 silencing in pDC specifically suppressed IFN-α production upon stimulation, providing a functional validation of our transfection protocol.

The discovery in the late 90’s of plasmacytoid dendritic cells (pDC) (also called Interferon-Producing Cells, IPC) profoundly increased our understanding of innate immune response^1^,^2^. At steady state, pDC are found at a low frequency in the thymus, peripheral lymphoid tissues and blood where they represent less than 1% of all peripheral blood mononuclear cells (PBMC)^3^. Nevertheless, they play a key role in the immune response to pathogens, and in particular to viruses, by producing very rapidly massive amounts of type I interferons (IFN), including all IFN-α isoforms and IFN-β^1^,^4^. Type I IFN secretion by pDC has an essential antiviral activity and major adjuvant functions on other immune cell-types^3^,^5^. To achieve pathogen sensing, pDC express the Toll-Like receptor TLR7 and TLR9^6^,^7^, which bind respectively single-stranded RNA^8^,^9^,^10^ and CpG-containing DNA^11^ molecules. Activation of pDC through TLR7 or TLR9 can trigger several types of response, but this essentially results in type I IFN production and/or differentiation into antigen-presenting cells. Two factors seem to be key for the induction of large quantities of type I IFN in pDC: 1) the ability of TLR ligands to bind their receptor in the early endosomal compartments^12^,^13^; 2) the phosphorylation and nuclear translocation of the transcription factor, the IFN regulatory factor 7 (IRF-7)^14^,^15^. However, molecular pathways leading to pDC activation are not fully understood as pDC are very rare, non-proliferating, fragile cells, which make functional studies a tough challenge^1^,^2^.

Since the discovery of RNA interference (RNAi), gene silencing using small interfering RNA (siRNA) has become a powerful functional genomics tool for studying gene function^16^,^17^. siRNA are double-stranded RNA molecules ranging from 19 to 25 nucleotides in length triggering sequence-specific mRNA degradation *via* a catalytic mechanism^18^. The high efficiency of RNAi methods to suppress the expression of specific genes in both cell lines and primary cells has revolutionized cell biology.

Moreover, major advances in siRNA modifications and delivery reagents have overcome initial problems of stability and cellular uptake in most cases. However, active cellular nucleases, specific membrane properties and other yet unidentified factors often make siRNA-mediated gene inactivation in human primary cells difficult^19^. Furthermore, some siRNA, also called immunostimulatory siRNA, can be endocytosed and thus trigger TLR7 pathway, leading to nonspecific pDC activation^20^,^21^. This phenomenon can be reduced by the incorporation of 2′-O-methyl modifications into the sugar structure of selected nucleotides within both the sense and antisense strands^21^,^22^. Such 2′-O-methyl modifications have also been shown to confer resistance to endonuclease activity^23^ and to abrogate off-target effects^24^. However, the transfection of siRNA in human primary pDC, in itself, remains a major challenge.

This latter issue was addressed in this report, as the transfection method seems to be decisive for a successful gene silencing. Among the various methods to deliver siRNA into cells, liposome-based systems are widespread due to their practical mode of use in most cell types. This method is based on siRNA packaging by cationic lipids into liposomal particles that facilitate the cellular uptake through plasma membrane and siRNA protection from enzymatic degradation during cellular endocytosis. Recently, liposome-based transfection has been shown particularly efficient for gene silencing in monocytes and myeloid dendritic cells^25^. Besides liposome-based technology, poly-ethylenimine (PEI) was one of the first transfection agent discovered^26^, after poly-L-lysine. PEI condenses nucleic acids into positively charged particles, which bind to anionic cell surface residues. Such polymer-oligonucleotide complexes (polyplex) are brought into the cell via endocytosis. Once inside the endosomes, protonation of the amines results in an influx of counter-ions and a lowering of the osmotic potential. Osmotic swelling bursts the endosomal vesicle, thus releasing the polyplex into the cytoplasm. If the polyplex unpacks, then the nucleic acids are free to diffuse to their targeted compartment^27^,^28^. However, PEI can be extremely cytotoxic^29^,^30^, due to the disruption of endosomal membranes leading to cellular stress and cell death. As an alternative to chemical agents, electroporation methods were also shown to have high transfection efficiencies in many cell lines. This method physically translocates siRNA into cells by a brief electric pulse, which induces a membrane perturbation allowing entry of nucleic acids. However, even if this technic is often used in primary human T cells, cells tend to exhibit higher levels of cell death after electroporation. Finally, gene silencing in hard-to-transfect cells can be achieved with virus-based vectors, and in particular lentiviruses, that encode short-hairpin RNA (shRNA) to induce specific mRNA degradation. However, pDC are known to be hardly infectable by Human Immunodeficiency Virus (HIV-1)^31^ and transduction with HIV-derived vectors might be inefficient. Furthermore, it is likely that RNA encapsidated within viral particles will engage TLR, triggering cellular activation and induce type I IFN production.

To our knowledge, there is no precise and efficient protocol dedicated to transfection and gene inactivation in primary human pDC by siRNA. In this report, we have evaluated a panel of six different methods to achieve gene silencing in pDC. Three different liposome-based reagents, including DOTAP, HiPerFect and the new generation of Lipofectamine RNAiMAX, an optimized PEI reagent (JetPRIME) and electroporation using the Amaxa Nucleofector technology were tested. Viral-based transduction using lentiviral vectors was also tested. We show that liposome-based method using DOTAP is the only efficient method for siRNA delivery into pDC, providing a technological solution for gene silencing and functional studies in this essential cellular population.

## Results

### Transfection and silencing efficiency in human primary pDC

**Figure 1:**
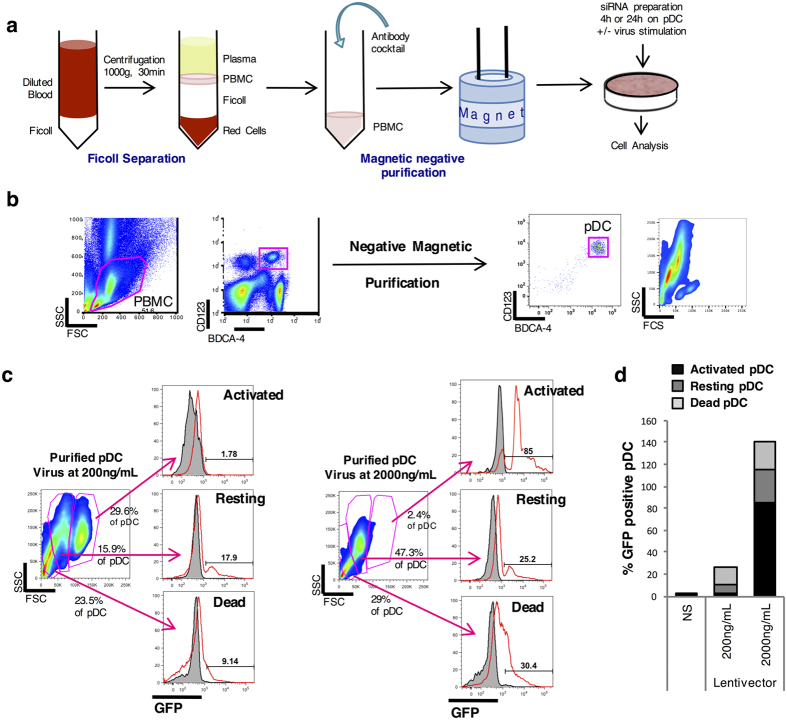
pDC transduction with a lentiviral vector is inefficient. (**a**) Illustration of pDC purification: PBMC are isolated from whole blood by Ficoll. Unwanted cells are targeted for removal with an antibody cocktail recognizing non-pDC (negative purification) and dextran-coated magnetic particles. The labeled cells are separated using the magnet. Desired cells are poured off into a new tube. Cells are cultured with the siRNA preparation the wanted time and stimulated or not overnight with various viruses. RNA, protein or supernatant are then analyzed. (**b**) Dot plot from cytometry represents whole PBMC then pDC after negative magnetic purification. (**c**) Purified pDC are put in culture with different concentrations of a VSV-G-pseudotyped GFP-encoding HIV-derived vector and transduction efficiency is evaluated by FACS 48 h post-infection. (**d**) Percentage of GFP positive purified pDC.

Human primary pDC were purified from peripheral blood of healthy donors by centrifugation on a density gradient and negative selection using antibody-coupled magnetic beads (Fig. 1a)^32^. Purity was assessed by flow cytometry analysis and was found superior to 90% (Fig. 1b). Cells were seeded at 10^5^ cells/mL in 96-well plates and treated with the different transfection methods as explained in Methods. First, we investigated whether a HIV-derived vector would be an appropriate tool to deliver a shRNA into pDC. In order to evaluate the transduction efficiency, we transduced pDC with a VSV-G-pseudotyped GFP-encoding HIV-derived vector at high MOI (200 ng/mL) and transduction efficiency was evaluated by FACS analysis. Only 17.9% of pDC expressed GFP, thus confirming that pDC are poorly transducible with HIV-derived vectors (Fig. 1c). Furthermore, we observed by confocal microscopy that pDC formed numerous giant mononuclear cells (GMC) (data not shown) at a 5–10 times higher frequency than previously described in HIV-treated pDC cultures^33^. Nevertheless, we tried to transduce pDC at a higher *multiplicity of infection* (MOI) in an attempt to increase the transduction efficiency. The activated pDC was defined as IKpDC, expressing TRAIL at its surface, as previously characterized^34^ (Supp. Fig. 1). pDC tend to change phenotype during the different stages of activation. The activating cells are bigger. The phenotype of the resting cells characterized the cells prior to their activation or prior to their death. However, when pDC were transduced at 2000 ng/mL, cell mortality reached 30% and the percentage of GFP-positive live cells was limited to 25% (Fig. 1c,d), even if a very small portion of cells (2.4%) were efficiently transduced. Altogether, these results disqualify this virus-based method to achieve gene silencing in pDC.

**Figure 2:**
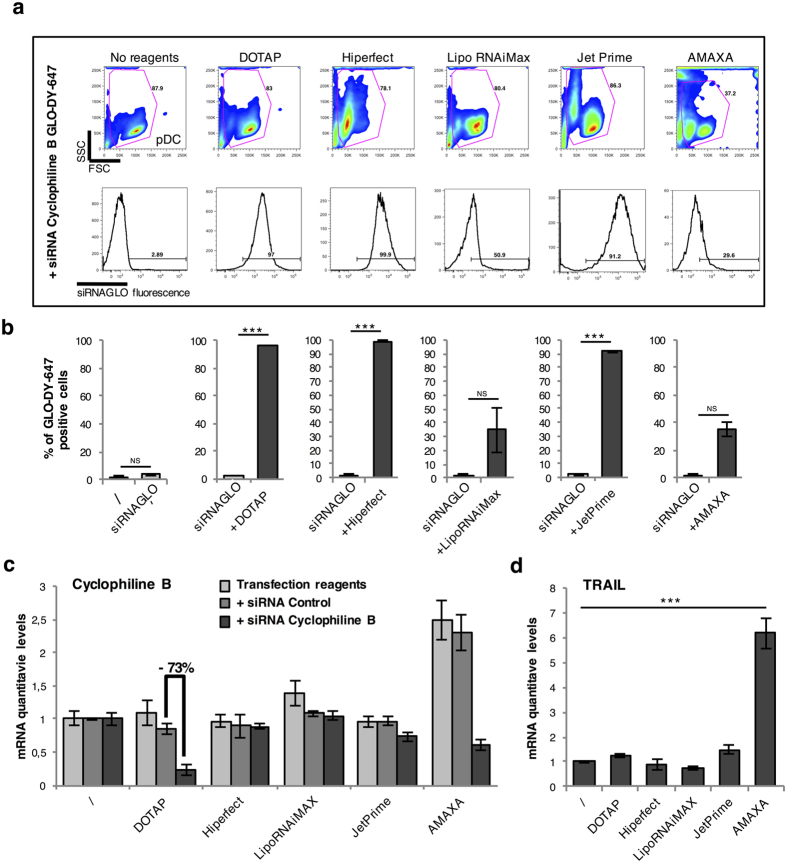
Comparison of the transfection efficiency of a fluorescent siRNA targeting cyclophilin B using the different transfection protocols. (**a**) Purified pDC were cultured for 4 hours with the different reagents complexed with the siRNA cyclophilin B coupled with siGLO-DY-547 so that the fluorescence of the siRNA could be measured by FACS (bottom panels) after evaluating the FSC/SSC (top panels). (**b**) The histograms represent the percentage of positive fluorescent cells, obtained as previously described in (**a**). (**c**) mRNA levels of cyclophilin B from purified pDC cultured for 24 hours with the different reagents alone, complexed with the siRNA Control or with the siRNA cyclophilin B were measured by RT-qPCR and normalized to RPL13A. (**d**) mRNA levels of TRAIL from purified pDC cultured for 24 hours with the different reagents complexed with the siRNA cyclophilin B were measured by RT-qPCR and normalized to RPL13A. ***P < 0.001, as calculated by one-way ANOVA with Tukey’s post hoc test. NS, not significant.

Then, we evaluated different chemical transfection reagents and electroporation to achieve pDC transfection with siRNA. As control for transfection, we used a siRNA targeting cyclophilin B that is labeled with the fluorescent dye DY-547 (siGLO-DY-547). The percentage of transfected pDC was determined by FACS analysis using fluorescence as readout (Fig. 2a,b). The siGLO was mixed with transfection agent DOTAP, HiPerFect, RNAiMAx or JetPRIME, and added to cell cultures following manufacturer’s instructions. For the Amaxa method, pDC were electro-stimulated following instructions adapted to primary DC cells. At 4 h post-transfection, a very high percentage of siGLO-DY-547 positive pDC was observed with DOTAP (96%), HiPerFect (98%) and JetPRIME (91%), suggesting some high transfection efficiency. In contrast, pDC transfected with Lipofectamine RNAiMAX or Amaxa showed lower percentages of transfection (35% and 30% of positive cells, respectively). We next tested the efficiency of gene silencing of the cyclophilin B gene in pDC using quantitative RT-PCR. As shown in Fig. 2c, mRNA levels of cyclophilin B where only reduced when pDC were transfected with DOTAP (more than 70%). In contrast, none of the other methods led to an efficient inhibition of cyclophilin B mRNA levels suggesting that DOTAP was the only efficient system to transfect pDC and achieve gene silencing. As the major issue with pDC transfection is cellular activation, this parameter was determined by quantification of TNF-related Apoptosis-Inducing Ligand (TRAIL) mRNA expression levels in pDC as previously described^35^,^36^. The siGLO complexed with either liposome-based transfection methods or JetPRIME did not induce any increase in TRAIL mRNA expression levels (Fig. 2d). In contrast, the Amaxa method induced 6-fold increase in TRAIL mRNA expression levels, thus supporting pDC activation by this transfection method.

### Viability and activation of pDC

**Figure 3:**
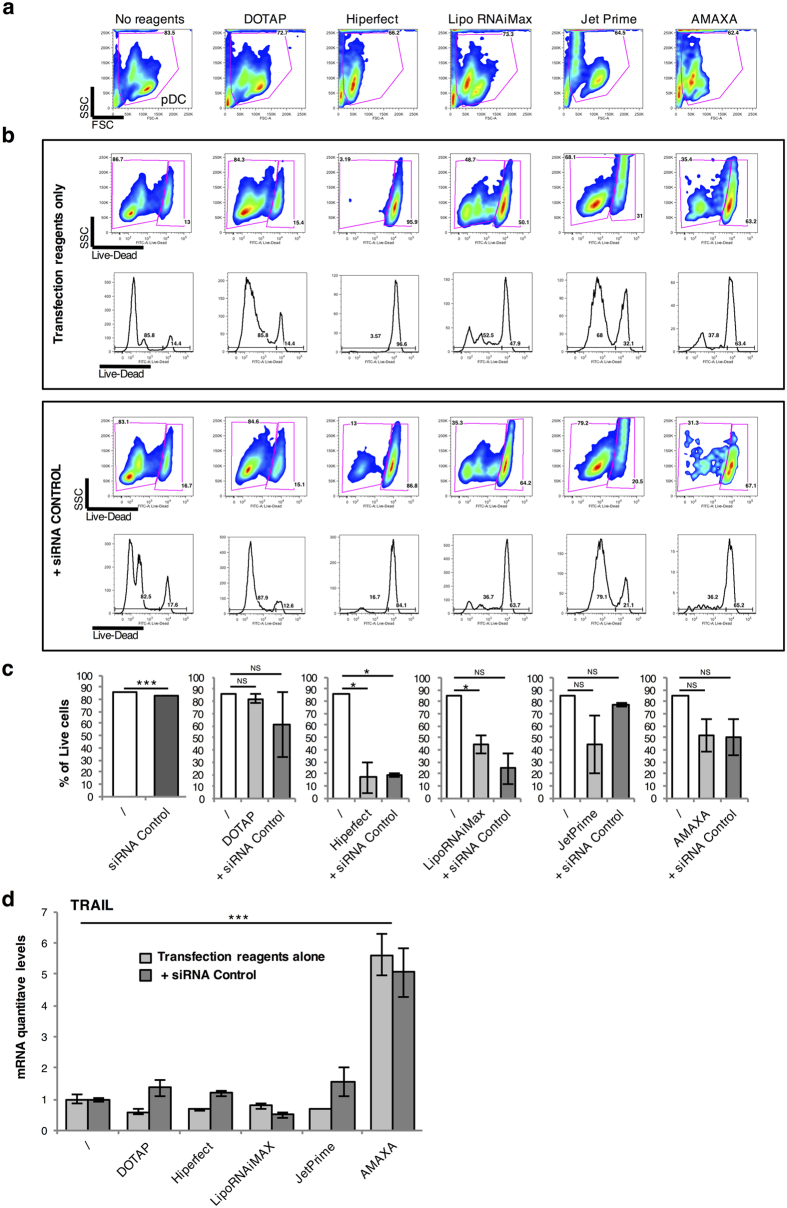
Comparison of the different transfection protocols (DOTAP, Hiperfect, LipoRNAiMax, JetPrime and Nucleofactor Amaxa) on pDC’s viability and activation. Purified pDC were cultured for 24 hours with the different reagents alone or complexed with the non-targeting siRNA Control. (**a**) The size (FSC) and granularity (SSC) of the cells were evaluated by FACS. (**b**) Purified pDC were stained with the LIVE/DEAD Green Dead Cell Stain Kit for 30min in order to evaluate the viability of the cells in presence of the reagents alone (top panels) or with the reagents complexed with the siRNA Control (lower panels). (**c**) The histograms represent the average of the percentage of live cells of 3 independent experiments. (**d**) mRNA levels of TRAIL from purified pDC cultured for 24 hours with the different reagents alone or complexed with the siRNA Control were measured by quantitative RT-PCR and normalized to RPL13A mRNA expression. *P < 0.05 and ***P < 0.001, as calculated by one-way ANOVA with Tukey’s post hoc test. NS, not significant.

In parallel to transfection efficiency, we determined the impact of the different protocols on pDC viability. Cells were subjected to the different methods of transfection in the absence or presence of control siRNA. Dead cells were stained specifically using the live-dead technology, and their percentage determined by flow cytometry analysis at 24 h post-treatment. As shown in Fig. 3b (upper panels), the percentage of living cells was similar in non-transfected and DOTAP-treated cells (around 85% of living cells). In contrast, the percentage of dead pDC massively increased when cultured in presence of HiPerFect or Lipofectamine RNAiMAX. Amaxa technology induced around 50% of cell death. However, the number of cells in the well was drastically reduced after the electroporation (loss of 70% of the cells) (Supp Fig. 1). Cell cultures treated with JetPRIME showed an intermediate percentage of dead cells, with approximately a two-fold increase compared to control. As shown in Fig. 3b (upper panels), similar results were obtained when treating cells with transfection methods in the presence of control siRNA (Fig. 3b – lower panels – and C).

We also evaluated whether transfection induced pDC activation, which was determined by quantification of TRAIL mRNA expression levels. Liposome-based transfection methods or JetPRIME did not induce any increase in TRAIL mRNA expression levels, whether control siRNA was present or not (Fig. 3d). In contrast, the Amaxa method induced 6-fold increase in TRAIL mRNA expression levels, as observed previously.

### Study of Transfection efficiency of labeled siRNA by microscopy

**Figure 4:**
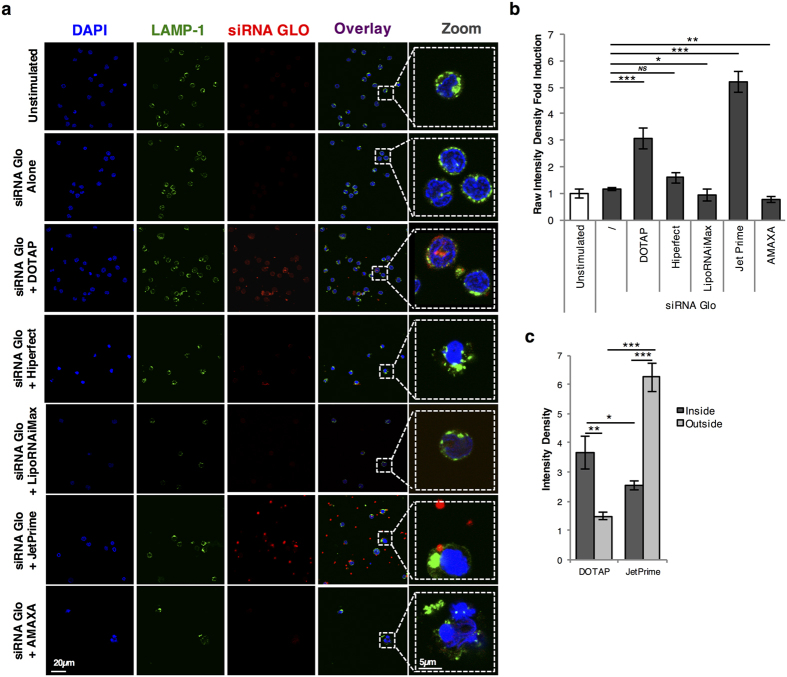
Subcellular localization of fluorescent siRNA targeting cyclophilin B using the different transfection protocols. (**a**) Purified pDC were cultured for 4 hours with the different reagents complexed with the siRNA cyclophilin B coupled with a GLO-DYE-547. Cells were harvested and coated on collagen slides, then fixed and permeabilized with saponin before being stained with LAMP1 (a late endosome marker). Cells were then mounted with Fluoromount G with DAPI. Scale bar, 20 μm or zoom at 5 μm. (**b**) The histograms represent the fold increase of the raw intensity density of the siRNA GLO measured with the JaCoP plugin in ImageJ, normalized to cells alone. (**c**) Intensity density of the fluorenscence of the siRNA GLO inside or outside the cells analyzed using the ImageJ software. *P < 0.05, **P < 0.01, and ***P < 0.001 as calculated by Student’s *t* test.

In order to better understand our observations, especially the high percentage of siGLO-DY-547 positive cells when transfected with JetPRIME contrasting with the very low level of gene silencing, we performed a microscopic study of pDC transfected with the various transfection reagents and especially, DOTAP and JetPRIME. Cells transfected with the siGLO-DY-547 were cultured for 4 h at 37 °C, then stained with both DAPI for nucleus labeling and the lysosomal-associated membrane protein 1, LAMP-1. In order to be efficient, siRNA should be localized in the cytoplasm but not into lysosomal compartments. As previously shown, pDC harbored a very large nucleus and a small cytoplasm characteristic of this cell subtype (Fig. 4a). When human pDC were incubated with the siGLO-DY-547 in the absence of any transfection reagent, we did not observe any siRNA associated to the cells, confirming that siRNA must be actively transfected to reach the intracellular compartment. In contrast, we found high levels of siRNA within pDC transfected with DOTAP (Fig. 4a,b). Furthermore, we showed that the labeled siRNA was not localized in lysosome, as we did not find any colocalization with the LAMP-1 marker. In fact, the vast majority of the siRNA was close to the nucleus. In contrast to results obtained with DOTAP, the vast majority of labeled siRNA transfected with JetPRIME was localized outside the cells (defined by localized in the medium or bound to the cell surface). We observed large aggregates of siRNA, forming large clusters of 0.5 to 2 μm diameter at the periphery of the cells. We thus quantified the “outside” siRNA versus the intracellular siRNA in cells transfected by DOTAP or JetPRIME. As shown in Fig. 4c, the vast majority of labeled siRNA was localized intracellularly when DOTAP was used, in contrast to JetPRIME transfected cells in which the siRNA was found outside the cells as large aggregates, either bound or not to cellular surface. This latest result may provide explanation for our previous flow cytometry and gene silencing efficiency data.

### Functional validation of gene silencing into human primary pDC

**Figure 5:**
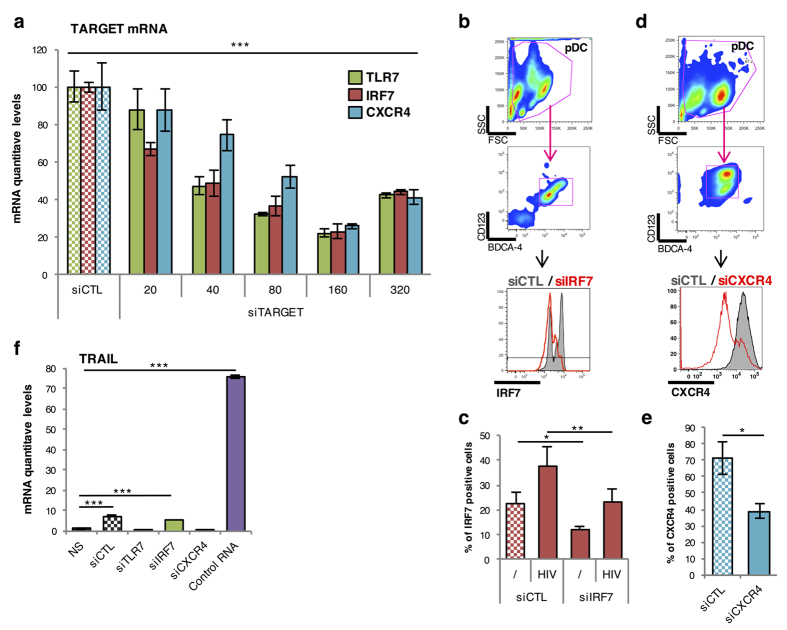
siRNA targeting TLR7, IRF7 and CXCR4 complexed with DOTAP inhibit the expression of the targeted mRNA and protein in human primary pDC. (**a**) mRNA levels of TLR7, IRF7 and CXCR4 (TARGET mRNA) from purified pDC cultured for 24 hours with different concentrations of siRNA Control (siCTL) or targeting (siTARGET) TLR7, IRF7 or CXCR4 complexed with DOTAP were measured by quantitative RT-PCR and normalized to RPL13A mRNA expression ***P < 0.0001, as calculated by two-way ANOVA. Concentration range was statistically different whereas the different siRNA treatments were not. (**b,d**) Protein level of IRF7 (**b**) or CXCR4 (**d**) from purified pDC cultured for 24 hours with siCTL or siIRF7 was evaluated by FACS. (**c**) Percentage of IRF7 positive cells treated with siCTL or siIRF7 stimulated or not with HIV overnight ***P < 0.001, as calculated Student’s *t* test. (**e**) Percentage of CXCR4 positive cells treated with siCTL or siCXCR4. *P < 0.05 as determined by standard Student’s *t* test (**f**) mRNA levels of TRAIL from untransfected purified pDC (NS) or transfected using DOTAP with the different siRNA (CTL, TLR7, IRF7 and CXCR4) at 160 nM or with a non-modified HIV-derived RNA were measured by quantitative RT-PCR and normalized to RPL13A mRNA expression. ***P < 0.001, as calculated by one-way ANOVA with Tukey’s post hoc test. NS, not significant.

**Figure 6:**
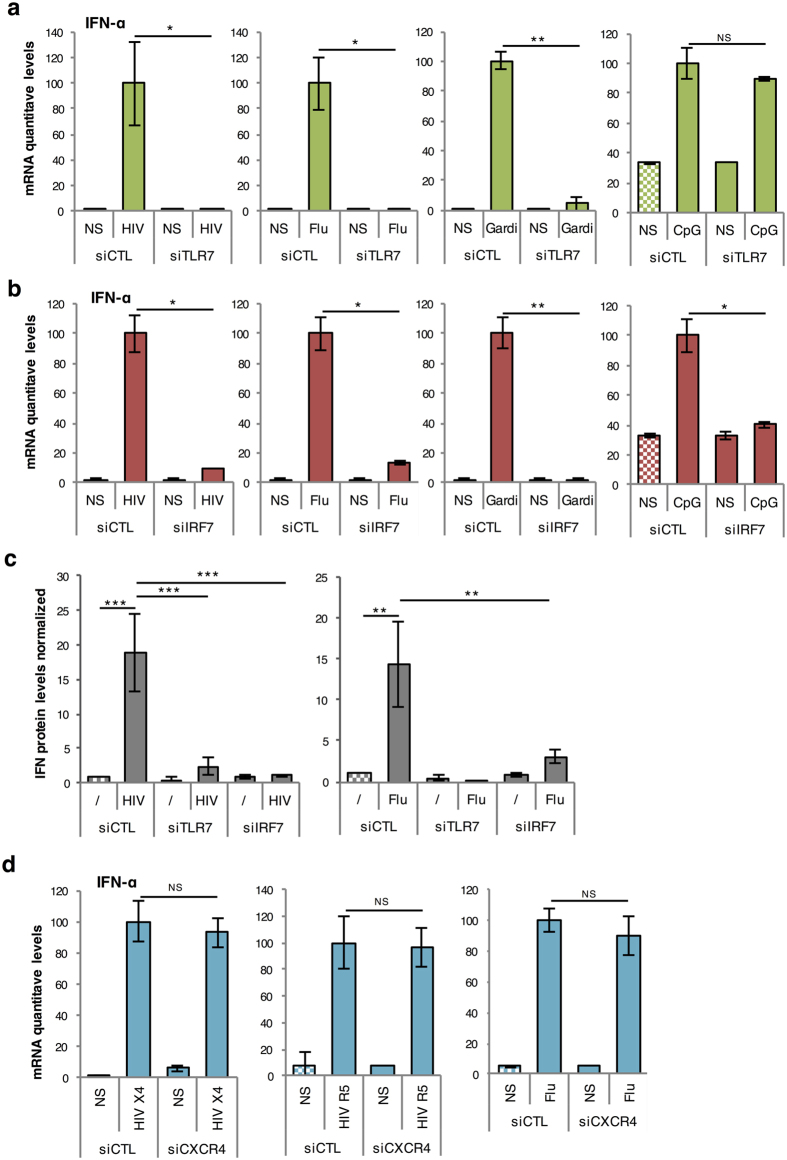
TLR7 or IRF7 silencing efficiently inhibits pDC activation. (**a,b**) mRNA levels of IFN-α from purified pDC cultured for 24 hours with DOTAP complexed with siRNA Control (siCTL), siTLR7 (**a**) or siIRF7 (**b**) and stimulated overnight with HIV (AT-2) X4, Influenza A virus, a TLR7 agonist Gardiquimod (Gardi) or the TLR9 ligand CpG_A_ were measured by quantitative RT-PCR and normalized to RPL13A mRNA expression. (**c**) Levels of IFN were measured in supernatants of purified pDC cultured for 4 hours with DOTAP complexed with siCTL or siIRF7 and stimulated overnight with HIV (AT-2) X4 or with Flu, using the STING-37 reporter cell line. Graphic is representative of three independent experiments. (**d**) mRNA levels of IFN-α from purified pDC cultured for 24 hours with DOTAP complexed with siRNA Control (siCTL) or siCXCR4 and stimulated overnight with HIV (AT-2) X4, HIV (AT-2) R5 or Influenza A virus were measured by quantitative RT-PCR and normalized to RPL13A. *P < 0.05, and **P < 0.01 as determined by standard Student’s *t* test. NS, not significant.

As key effector cells of the innate immune response, pDC express TRAIL and produce massive amounts of type I IFN after recognition by TLR7 of single-stranded RNA from pathogens such as HIV or Influenza A. This TLR7 activation induces the phosphorylation and nuclear translocation of IRF-7, which is the main transcription factor regulating type I IFN production in pDC^14^,^37^. We thus attempted to silence TLR7 and IRF-7 in human primary pDC by siRNA transfection with DOTAP. As a negative control, we decided to silence the C-X-C chemokine receptor type 4 (CXCR4), a highly expressed chemokine receptor that is not implicated in TRAIL or type I IFN production in HIV-stimulated pDC^36^,^38^. We first tested several concentrations of TLR7, IRF-7, or CXCR4 siRNA, and showed that 160 nM was the optimal concentration for an efficient silencing of targeted mRNA (Fig. 5a). This was confirmed at protein levels, as assessed by IRF-7 or CXCR4 immunostaining and FACS analysis (TLR7 protein could not be determined as we are missing a specific antibody) (Fig. 5b–e). We then verified that at this concentration, TLR7, IRF-7 and CXCR4 siRNAs were not inducing pDC activation. For this, we cultured pDC for 24 h with one of the four siRNA (CTL, TLR7, IRF-7 or CXCR4) or with a synthetic non-modified viral RNA (35 nucleotides long), all complexed with DOTAP at the concentration 160 nM and we measured TRAIL mRNA production by quantitative RT-PCR. As shown in Fig. 5f, none of the siRNA induced pDC activation at 160 nM unlike the control RNA, which induced a dramatic increase in TRAIL mRNA expression. Finally, we showed that TLR7 or IRF-7 siRNA drastically reduced IFN-α induction in pDC stimulated with TLR7 activators, including HIV-1, influenza virus and Gardiquimod (Fig. 6a,b). In contrast and as expected, IRF7, but not TLR7 silencing inhibited IFN-α when pDC were activated with the TLR9 ligand CpG_A_ (Fig. 6a,b). We also showed by using a functional cellular assay that type I IFN secretion was strongly inhibited in pDC treated with TLR7 or IRF-7 siRNA (Fig. 6c). In contrast, CXCR4 silencing had not effect on IFN-α transcription, thus demonstrating that DOTAP transfected pDC were perfectly functional and produced similar levels of cytokine compared to untransfected cells (Fig. 6d). Altogether, these results demonstrate that gene silencing can be achieved in pDC by siRNA transfection with DOTAP, while preserving cellular viability and functional properties (Table 1).

## Discussion

In this report, we have established a protocol for gene silencing in human primary pDC, a key effector cell type involved in immune response to pathogens and autoimmunity^34^,^39^,^40^,^41^. The method described herein opens new perspectives for the functional study of these cells. Primary pDC are extremely rare and fragile cells that only survive a few days in cell culture and do not proliferate, making their transfection with siRNA a real challenge. Some reports have previously described gene silencing in bone marrow-derived mouse pDC or pDC-related cells. The GeneSilencer siRNA Transfection Reagent (a cationic liposome reagent) or the Amaxa technology were previously used to achieve gene silencing in murine pDC derived *in vitro* from proliferating bone marrow precursors^23^. It was also shown that a lentiviral vector coding for a specific shRNA could silence TLR7 expression in Gen2.2 cells, a human cell line that is often used as a surrogate of human pDC. As a result, IFN response to HIV, foamy viruses or influenza A virus was impaired^42^,^43^. Zhang & al. developed siRNA linked to CpG oligonucleotides to target TLR9 and facilitate siRNA internalization. Such siRNA-CpG conjugates were successfully used to silence STAT3 in monocyte-derived CD303 + (BDCA-2) cells that exhibit phenotypic similarities with pDC^44^. However, and to our knowledge, our report is the first to achieve gene silencing in primary human pDC.

We established that most transfection methods tested, including virus-like particles, HiPerFect, Lipofectamine RNAiMAX and Amaxa, induced massive cell death of primary pDC. JetPRIME was not highly cytotoxic, but formed large aggregates that remained in the extracellular compartment and failed to deliver siRNA into the cells. Only DOTAP was found efficient to deliver siRNA in the cytoplasm where gene silencing is achieved. Reasons why DOTAP was efficient and not cytotoxic, compared to other liposome-based reagents like HiPerFect or RNAiMAX, remain elusive. We can only assume that compared to HiPerFect or Lipofectamine RNAiMAX, DOTAP transfection reagent, which is based on 1,2-dioleoyl-3-trimethylammonium-propane monocationic lipid, is the best compromise in terms of positive charges and diameter of liposome particles to transfect pDC. We also showed that none of the siRNA tested induced pDC activation by TLR7 and this probably relates to siRNA modifications. In this report we used the ON-TARGET*plus* siRNA reagents from Dharmacon, which uses the SMARTpool technology and 3 of 4 individual siRNA to target the mRNA. Furthermore, these siRNA have been 2′-*O*-methyl modified, which was previously reported to decrease off-target activity and cell activation^24^,^45^. Finally, we determined that the best siRNA concentration for gene silencing in pDC (160 nM) is very close to the one successfully used in primary human monocytes and conventional dendritic cells (200 nM)^25^.

This transfection protocol allowed cyclophilin B, CXCR4, IRF-7 and TLR7 efficient silencing in primary pDC. Cyclophilin B or CXCR4 silencing had no impact on the cellular response to TLR7 ligands, thus demonstrating that siRNA transfection with DOTAP did not alter IFN-mediated pDC production. In contrast, TLR7 or IRF-7 silencing impaired pDC response to TLR7 ligands, whereas only IRF-7 silencing also inhibited pDC activation by a TLR9 ligand. It was previously shown in TLR7 knock-out mice^8^, that pDC response to single-stranded RNA depends on TLR7. Here, we provide some direct demonstration that TLR7 is required for the sensing of HIV or influenza virus particles by primary human pDC. We also confirmed that IRF-7 is needed, something previously established in primary human pDC from patients with mutated forms of this transcription factor^37^. Altogether, this provides a robust validation of our transfection protocol at the mRNA and protein levels that should greatly facilitate functional studies of this unique cell type.

## Methods

### Blood samples, isolation and culture of blood leukocytes

Blood from healthy HIV-1-seronegative blood bank donors was obtained from *“Etablissement Français du Sang”* (convention # 07/CABANEL/106), Paris, France. A written informed consent was obtained from all subjects involved. Experimental procedures with human blood have been approved by Necker Hospital Ethical Committees for human research and were done according to the European Union guidelines and the Declaration of Helsinki. *In vitro* experiments were performed using human peripheral blood mononuclear cells (PBMC) isolated by density centrifugation with Lymphoprep medium (StemCell Technologies). pDC were purified by negative selection with the Human plasmacytoid DC enrichment kit (StemCell Technologies). Cells were cultured in RPMI 1640 (Invitrogen, Gaithersburg, MD) containing 10% fetal bovine serum (Hyclone, Logan, UT). After purification, we obtained purity higher than 90% for pDC.

### Viral stimulation and infection

Purified pDC were seeded at 10^5^/100 μl and then stimulated with the following viruses: inactivated aldrithiol-2 (AT-2) HIV-1_MN_ (CXCR4 co-receptor specific) or AT-2 HIV-1_ADA_ (CCR5 co-receptor specific) at 60 ng/mL p24^CA^ equivalent (kindly provided by J.D. Lifson: SAIC-NCI, Frederick, MD), infectious human Influenza A/PR/8/34 virus (Flu), titer 1:8192 at dilution 1:1000, the TLR7 specific synthetic activator Gardiquimod at 10 μg/mL or the TLR9 specific synthetic activator CpG_A_ at 5 μM.

Vesicular stomatitis virus glycoprotein (VSV-G)-pseudotyped HIV-1-derived vectors were generated by cotransfecting HEK293T cells with pVSV-G, a HIV-1 Gag-Pol expression plasmid (p8.91) and green fluorescent protein (GFP)-expressing lentiviral vector, using the ProFection calcium phosphate kit (Promega). Lentiviral vectors were purified using Lenti-X Concentrator (Takara Clontech) and titrated by p24 ELISA (Lenti-X p24 Rapid Titer Kit, Takara Clontech). Purified pDC were cultured for 48 h in presence of lentiviral particles. Supernatants were collected for cytokine detection. Microvesicles isolated from uninfected cell cultures matched to the culture to produce the virus were used as negative control (Mock).

### Small interference RNA experiments

pDCs were seeded at 10^5^ cells/100 μL in 96-well plates and incubated at 37 °C. The ON-TARGETplus SMARTpool siRNA targeting TLR7, IRF-7 or CXCR4 as well as the ON-TARGETplus Non-targeting siRNA (siCTL) and the siGLO Cyclophilin B Control siRNA were purchased from Thermo Scientific, Dharmacon (Illkirch, France). Upon arrival, siRNAs came in dried pellets and were stored as such at −20 °C. The resuspension of siRNA pellets was done in 1X siRNA Buffer (Thermo Scientific, Dharmacon). The working aliquots (20 μM) were then stored at −20  °C. In this report, we used a final siRNA concentration of 160 nM: 0.88 μl (=17.6 pmol or ≈0.23 μg) for the six-well plates from the 20 μM siRNA working stocks. Each transfection agent was used according to manufacturer’s protocol. Briefly:

For DOTAP, the right volume of siRNA for a final concentration of 160 nM was diluted in PBS (1:5) then DOTAP (Roche Applied Sciences) was added v/v. The mix was incubated at room temperature during 15 minutes and then was added to cells in culture (RPMI-10% FBS).

For Hiperfect, the right volume of siRNA for a final concentration of 160 nM was mix with 6 μL of Hiperfect (Qiagen) and 85μL of X-*vivo* 15 media (Lonza) for 5 to 10 minutes at room temperature before the cells were added to the mix and put in culture.

For Lipofectamine RNAiMAX, the right volume of siRNA for a final concentration of 160 nM was diluted in 50 μL of X-*vivo* 15 media. The Lipofectamine RNAiMAX reagent (1 μL) (Thermo Fisher Scientific) was diluted in 50 μL of X-*vivo* 15 medium for 5 minutes at room temperature. The two solutions were mixed and incubated for 20 minutes at room temperature before the cells were added to the mix and put in culture.

For JetPrime, the right volume of siRNA for a final concentration of 160 nM was diluted in 12,5 μL of JetPrime Buffer and 2 μL of JetPrime (Polyplus) were added. The mix was incubated at room temperature during 10 minutes and then was added to cells in culture (RPMI-10% FBS).

For Amaxa, we used the Amaxa Human Dendritic Cell Nucleofector Kit and Amaxa II Nucleofactor (Lonza). The supplement was added to the nucleofector solution. The cell’s pellet was resuspend in 100 μL of mix and the right volume of siRNA for a final concentration of 160 nM was added. We used the Nucleofector^®^Program U-002 program. Once the electroporation over, the cells were centrifuged to get rid of all supernatant and resuspend in 100 μL of media (RPMI-10% FBS).

A synthetic non-modified viral RNA (35 nucleotides long) was used as a control RNA and complexed with DOTAP with the same method describe previously.

Finally, cells were incubated at 37 °C for 24 hours before adding the different virus overnight.

### Flow Cytometry

Cultured cells were incubated for 20 min at 4 °C with appropriate antibodies APC-conjugated anti-BDCA-4, FITC-anti-CD123 (Miltenyi, Bergisch Gladbach, Germany), PE.cy7-anti-CXCR4 clone 12G5 (Biolegend, San Diego, CA), Live/Dead Green Kit (ThermoFisher Scientific) or with appropriate isotype-matched control antibodies (5μg/mL each) in PBS containing 2% mouse serum (Sigma, Saint Louis, MO) and FC-receptor blockers (BD Biosciences, San Jose, CA). For IRF-7 intracellular staining, cells were fixed with 2% PFA then permeabilized with 0.5% saponin before being stained for 30 minutes at 4 °C with anti-IRF-7 antibody (BD Biosciences). Flow cytometry analysis was performed on a flow cytometry Canto II flow cytometer using flow cytometry Diva software (BD Biosciences, San Jose, CA). FlowJo software (Treestar, Ashland, OR) was used to analyze data.

### Three dimensional microscopy

Purified pDC cells cultured 4 h in presence of siRNA GLO-DY-547 complexed with the different transfection reagents. pDC cells were plated on collagen (ThermoFisher Scientific)-coated slides and then fixed in 4% paraformaldehyde, quenched with 0.1 M glycine. Cells were incubated in permeabilizing buffer containing 0.5% saponin with monoclonal antiboby anti-LAMP-1 (Cell Signaling). LAMP-1 was revealed by a Goat anti-rat-Alexa Fluor 594 (LifeTechnologies). Slides were mounted with DAPI Fluoromount-G (SouthernBiotech). Slides were scanned with a Confocal Microscope Zeiss LSM 710 (Carl Zeiss Microscopy) using a 63x Plan-Apochromat objective and images were acquired with the Zen software. Images of purified pDC were analyzed using the ImageJ software (NIH, Bethesda, MD, USA).

### Type I IFN detection

To quantify the secretion of functional IFN-alpha in pDC cultures, we used a biological assay based on a stable cell line where luciferase reporter gene is controlled by five Interferon-Stimulated Response Elements (STING37 cell line^46^). First, pDC supernatants were harvested after 24 h of stimulation and frozen at −20 °C for storage. Then, serial dilutions of pDC supernatants were dispensed in culture wells of a 96-well plate containing 35.10^3^ STING37 cells per well. After 24 h of culture, luciferase activity was determined by adding 50 μL of Bright-Glo reagents (Promega) to culture wells and measuring bioluminescence with a luminometer. Presented data correspond to the dilution point where virtually IFN-alpha activity was detected in supernatants of unstimated pDC.

### RT-qPCR analyses

Total RNA was extracted using RNeasy Mini kit and was submitted to DNase treatment (Qiagen), following manufacturer’s instructions. RNA samples were converted to cDNA with RevertAid H Minus First Strand cDNA Synthesis Kit (Thermo Scientific). Real-time PCR reactions were performed in duplicates using Takyon ROX SYBR MasterMix blue dTTP (Eurogentec) on a 7900HT Fast Real-Time PCR System (Applied Biosystems). Transcripts were quantified using the following program: 3 min at 95 °C followed by 35 cycles of 15 s at 95 °C, 25 s at 60 °C and 25 s at 72 °C. Values for each transcript were normalized to expression levels of RPL13A (60S ribosomal protein L13a) using the 2-ΔΔCt method. Primers used for quantification of transcripts by real time quantitative PCR are indicated below in Table 2:

### Statistical analysis

P values (*P*) were determined using Student’s *t* test, one-way ANOVA or two-way ANOVA and stated in each figure legend. *P* < 0.05 was considered statistically significant. *P < 0.05; **P < 0.01 and ***P < 0.001. Univariate distributions of flow cytometry data were performed by probability binning, in 300 bins using FlowJo software.

## Additional Information

**How to cite this article**: Smith, N. *et al*. An efficient method for gene silencing in human primary plasmacytoid dendritic cells: silencing of the TLR7/IRF-7 pathway as a proof of concept. *Sci. Rep.* **6**, 29891; doi: 10.1038/srep29891 (2016).

## Supplementary Material

Supplementary Information

## Figures and Tables

**Table 1:** Overview of transfection protocol’s features.

	**+DOTAP**	**+Hiperfect**	**+LipoRNAiMAX**	**+JetPrime**	**+AMAXA**
Live cells (Transfection reagent alone)	**++**	**− −**	**− −**	**− −**	**−**
Live cells (+siRNA Control)	**++**	**− −**	**+**	**− − −**	**+**
Transfection Efficiency	**+++**	**+++**	**+**	**+++**	**++**
Targeted mRNA inhibition	**+++**	**− −**	**− −**	**−**	**+**
Absence of cell activation	**++**	**++**	**++**	**−**	**− − −**
**Silencing efficiency score**	**+++**	**− −**	**−**	**− − −**	**−**

The table summarizes the effects of each transfection protocol complexed or not with siRNA. Legend: “Live cells”: “+” represents live cells; “Transfection Efficiency”: “+” represents a high transfection efficiency; “Targeted mRNA inhibition”: “+” represents downregulation of the mRNA targeted by the siRNA; “Absence of cell activation”: “+” represents no cell activation.

**Table 2:** Primer’s sequences.

**Gene**	**Forward Primer sequence (5′ −>3′)**	**Reverse Primer sequence (5′−>3′)**	**Size**
RPL13A	CTGGAACGGTGAAGGTGACA	TTGAGGACCTCTGTGTATTTGTCAA	126 bp
TRAIL	GCTGAAGCAGATGCAGGACAA	TGACGGAGTTGCCACTTGACT	135 bp
CypB	GAACGCAACATGAAGGTGCTC	CCCCTTCTTCTTCTCATCGGC	102 bp
IFN-α1/13^1^	CCAGTTCCAGAAGGCTCCAG	TCCTCCTGCATCACACAGGC	174 bp
TLR7	CTAAAGACCCAGCTGTGACCGA	CCAGTCCCTTTCCTCGAGACAT	104 bp
IRF-7	CAGATCCAGTCCCAACCAAG	GTCTCTACTGCCCACCCGTA	118 bp
CXCR4	GCATGACGGACAAGTACAGGCT	AAAGTACCAGTTTGCCACGGC	101 bp

^1^Primers amplify both IFN-α1 and IFN-α13 transcripts.

## References

[b1] Siegal F. P. . The nature of the principal type 1 interferon-producing cells in human blood. Science 284, 1835–1837 (1999). 10364556 10.1126/science.284.5421.1835

[b2] Grouard G. . The enigmatic plasmacytoid T cells develop into dendritic cells with interleukin (IL)-3 and CD40-ligand. J Exp Med 185, 1101–1111 (1997). 9091583 10.1084/jem.185.6.1101 PMC2196227

[b3] Colonna M., Trinchieri G. & Liu Y. J. Plasmacytoid dendritic cells in immunity. Nat Immunol 5, 1219–1226 (2004). 15549123 10.1038/ni1141

[b4] Cella M. . Plasmacytoid monocytes migrate to inflamed lymph nodes and produce large amounts of type I interferon. Nat Med 5, 919–923 (1999). 10426316 10.1038/11360

[b5] Yang G. X. . Plasmacytoid dendritic cells of different origins have distinct characteristics and function: studies of lymphoid progenitors versus myeloid progenitors. J Immunol 175, 7281–7287 (2005). 16301633 10.4049/jimmunol.175.11.7281

[b6] Jarrossay D., Napolitani G., Colonna M., Sallusto F. & Lanzavecchia A. Specialization and complementarity in microbial molecule recognition by human myeloid and plasmacytoid dendritic cells. Eur J Immunol 31, 3388–3393 (2001). 11745357 10.1002/1521-4141(200111)31:11<3388::aid-immu3388>3.0.co;2-q

[b7] Kadowaki N. & Liu Y. J. Natural type I interferon-producing cells as a link between innate and adaptive immunity. Hum Immunol 63, 1126–1132 (2002). 12480256 10.1016/s0198-8859(02)00751-6

[b8] Diebold S. S., Kaisho T., Hemmi H., Akira S. & Reis e Sousa C. Innate antiviral responses by means of TLR7-mediated recognition of single-stranded RNA. Science 303, 1529–1531 (2004). 14976261 10.1126/science.1093616

[b9] Heil F. . Species-specific recognition of single-stranded RNA via toll-like receptor 7 and 8. Science 303, 1526–1529 (2004). 14976262 10.1126/science.1093620

[b10] Lund J. M. . Recognition of single-stranded RNA viruses by Toll-like receptor 7. Proc Natl Acad Sci USA 101, 5598–5603 (2004). 15034168 10.1073/pnas.0400937101 PMC397437

[b11] Hemmi H. . A Toll-like receptor recognizes bacterial DNA. Nature 408, 740–745 (2000). 11130078 10.1038/35047123

[b12] Guiducci C. . Properties regulating the nature of the plasmacytoid dendritic cell response to Toll-like receptor 9 activation. The Journal of experimental medicine 203, 1999–2008 (2006). 16864658 10.1084/jem.20060401 PMC2118381

[b13] Honda K. . Spatiotemporal regulation of MyD88-IRF-7 signalling for robust type-I interferon induction. Nature 434, 1035–1040 (2005). 15815647 10.1038/nature03547

[b14] Honda K. . IRF-7 is the master regulator of type-I interferon-dependent immune responses. Nature 434, 772–777 (2005). 15800576 10.1038/nature03464

[b15] Guiducci C. . PI3K is critical for the nuclear translocation of IRF-7 and type I IFN production by human plasmacytoid predendritic cells in response to TLR activation. J Exp Med 205, 315–322 (2008). 18227218 10.1084/jem.20070763 PMC2271003

[b16] Hamilton A. J. & Baulcombe D. C. A species of small antisense RNA in posttranscriptional gene silencing in plants. Science 286, 950–952 (1999). 10542148 10.1126/science.286.5441.950

[b17] Bernstein E., Caudy A. A., Hammond S. M. & Hannon G. J. Role for a bidentate ribonuclease in the initiation step of RNA interference. Nature 409, 363–366 (2001). 11201747 10.1038/35053110

[b18] Elbashir S. M. . Duplexes of 21-nucleotide RNAs mediate RNA interference in cultured mammalian cells. Nature 411, 494–498 (2001). 11373684 10.1038/35078107

[b19] Martinez F. O. Analysis of gene expression and gene silencing in human macrophages. Curr Protoc Immunol Chapter 14, Unit 14 28 11–23 (2012). 22314831 10.1002/0471142735.im1428s96

[b20] Judge A. D. . Sequence-dependent stimulation of the mammalian innate immune response by synthetic siRNA. Nat Biotechnol 23, 457–462 (2005). 15778705 10.1038/nbt1081

[b21] Hornung V. . Sequence-specific potent induction of IFN-alpha by short interfering RNA in plasmacytoid dendritic cells through TLR7. Nat Med 11, 263–270 (2005). 15723075 10.1038/nm1191

[b22] Judge A. D., Bola G., Lee A. C. & MacLachlan I. Design of noninflammatory synthetic siRNA mediating potent gene silencing *in vivo*. Mol Ther 13, 494–505 (2006). 16343994 10.1016/j.ymthe.2005.11.002

[b23] Prete F. . Wiskott-Aldrich syndrome protein-mediated actin dynamics control type-I interferon production in plasmacytoid dendritic cells. J Exp Med 210, 355–374 (2013). 23337808 10.1084/jem.20120363 PMC3570108

[b24] Jackson A. L. . Position-specific chemical modification of siRNAs reduces “off-target” transcript silencing. Rna 12, 1197–1205 (2006). 16682562 10.1261/rna.30706 PMC1484422

[b25] Troegeler A. . An efficient siRNA-mediated gene silencing in primary human monocytes, dendritic cells and macrophages. Immunology and cell biology (2014). 10.1038/icb.2014.39 24890643

[b26] Boussif O. . A versatile vector for gene and oligonucleotide transfer into cells in culture and *in vivo*: polyethylenimine. Proc Natl Acad Sci USA 92, 7297–7301 (1995). 7638184 10.1073/pnas.92.16.7297 PMC41326

[b27] Rudolph C., Lausier J., Naundorf S., Muller R. H. & Rosenecker J. *In vivo* gene delivery to the lung using polyethylenimine and fractured polyamidoamine dendrimers. J Gene Med 2, 269–278 (2000). 10953918 10.1002/1521-2254(200007/08)2:4<269::AID-JGM112>3.0.CO;2-F

[b28] Akinc A., Thomas M., Klibanov A. M. & Langer R. Exploring polyethylenimine-mediated DNA transfection and the proton sponge hypothesis. J Gene Med 7, 657–663 (2005). 15543529 10.1002/jgm.696

[b29] Moghimi S. M. . A two-stage poly(ethylenimine)-mediated cytotoxicity: implications for gene transfer/therapy. Mol Ther 11, 990–995 (2005). 15922971 10.1016/j.ymthe.2005.02.010

[b30] Hunter A. C. Molecular hurdles in polyfectin design and mechanistic background to polycation induced cytotoxicity. Adv Drug Deliv Rev 58, 1523–1531 (2006). 17079050 10.1016/j.addr.2006.09.008

[b31] Lederle A. . Neutralizing antibodies inhibit HIV-1 infection of plasmacytoid dendritic cells by an FcgammaRIIa independent mechanism and do not diminish cytokines production. Scientific reports 4, 5845 (2014). 25132382 10.1038/srep05845 PMC4135332

[b32] Megjugorac N. J., Gallagher G. E. & Gallagher G. Modulation of human plasmacytoid DC function by IFN-lambda1 (IL-29). J Leukoc Biol 86, 1359–1363 (2009). 19759281 10.1189/jlb.0509347

[b33] Smith N., Etheve-Quelquejeu M. & Herbeuval J. P. Transformation of Plasmacytoid Dendritic Cells into Giant Multinuclear Cells by HIV-1. AIDS Res Hum Retroviruses (2015). 10.1089/aid.2015.0082 26060879

[b34] Hardy A. W., Graham D. R., Shearer G. M. & Herbeuval J. P. HIV turns plasmacytoid dendritic cells (pDC) into TRAIL-expressing killer pDC and down-regulates HIV coreceptors by Toll-like receptor 7-induced IFN-alpha. Proc Natl Acad Sci USA 104, 17453–17458 (2007). 17956986 10.1073/pnas.0707244104 PMC2077277

[b35] Barblu L. . Plasmacytoid Dendritic Cells (pDCs) From HIV Controllers Produce Interferon-alpha and Differentiate Into Functional Killer pDCs Under HIV Activation. J Infect Dis 206, 790–801 (2012). 22693234 10.1093/infdis/jis384

[b36] Smith N., Etheve-Quelquejeu M. & Herbeuval J. P. CD4 and Tumor Necrosis Factor-Related Apoptosis Ligand (TRAIL) localization in HIV-stimulated plasmacytoid dendritic cells by three-dimensional microscopy. AIDS Res Hum Retroviruses 30, 1158–1159 (2014). 25343561 10.1089/aid.2014.0125

[b37] Ciancanelli M. J. . Infectious disease. Life-threatening influenza and impaired interferon amplification in human IRF7 deficiency. Science 348, 448–453 (2015). 25814066 10.1126/science.aaa1578 PMC4431581

[b38] Pritschet K. . CD4- and dynamin-dependent endocytosis of HIV-1 into plasmacytoid dendritic cells. Virology 423, 152–164 (2012). 22209232 10.1016/j.virol.2011.11.026

[b39] Barchet W., Cella M. & Colonna M. Plasmacytoid dendritic cells–virus experts of innate immunity. Semin Immunol 17, 253–261 (2005). 15990333 10.1016/j.smim.2005.05.008

[b40] Pulendran B., Palucka K. & Banchereau J. Sensing pathogens and tuning immune responses. Science 293, 253–256 (2001). 11452116 10.1126/science.1062060

[b41] Banchereau J., Pascual V. & Palucka A. K. Autoimmunity through cytokine-induced dendritic cell activation. Immunity 20, 539–550 (2004). 15142523 10.1016/s1074-7613(04)00108-6

[b42] Rua R., Lepelley A., Gessain A. & Schwartz O. Innate sensing of foamy viruses by human hematopoietic cells. J Virol 86, 909–918 (2012). 22090096 10.1128/JVI.06235-11 PMC3255846

[b43] Lepelley A. . Innate sensing of HIV-infected cells. PLoS Pathog 7, e1001284 (2011). 21379343 10.1371/journal.ppat.1001284 PMC3040675

[b44] Zhang Q. . TLR9-mediated siRNA delivery for targeting of normal and malignant human hematopoietic cells *in vivo*. Blood 121, 1304–1315 (2013). 23287859 10.1182/blood-2012-07-442590 PMC3578952

[b45] Eberle F. . Modifications in small interfering RNA that separate immunostimulation from RNA interference. J Immunol 180, 3229–3237 (2008). 18292547 10.4049/jimmunol.180.5.3229

[b46] Lucas-Hourani M. . Inhibition of pyrimidine biosynthesis pathway suppresses viral growth through innate immunity. PLoS Pathog 9, e1003678 (2013). 24098125 10.1371/journal.ppat.1003678 PMC3789760

