# Supplementary material for: An efficient method for gene silencing in human primary plasmacytoid dendritic cells: silencing of the TLR7/IRF-7 pathway as a proof of concept
Source: Sci Rep. 2016 Jul 14;6:29891. doi: 10.1038/srep29891 (PMC4944138; doi:10.1038/srep29891)
Supplement: Supplementary Information [file srep29891-s1.doc]

**An efficient method for gene silencing in human primary plasmacytoid dendritic cells: silencing of the TLR7/IRF‑7 pathway as a proof of concept**

Nikaïa Smith^1,2^, Pierre‑Olivier Vidalain^1,2^, Sébastien Nisole^2,3^, Jean‑Philippe Herbeuval^1,2^

**Supplemental figureS**

**Supplemental Figure 1**

**Supplemental Figure 1:** **The live activated purified pDC highly express the pDC activation marker TRAIL**. The level of the pDC activation surface marker TNF-Related Apoptosis Ligand (TRAIL) was assessed by FACS on purified pDC stimulated or not with HIV. The dot plots on the left show the size (FCS) and granularity (SSC) of the pDC. The histograms in the middle show the levels of TRAIL on each subset of pDC (activated, resting and dead cells). The histogram on the right shows the total level of TRAIL.

**Supplemental Figure 2**

**Supplemental Figure 2:** **Quantification of the number of cells after transfection**. The number of cells per well were counted by FACS after 4h with the different siRNA treatments (transfection reagent with or without siRNA) and normalized to the non stimulated well (NS).
